# Factors Influencing Mortality in Children with Central Nervous System Tumors: A Cohort Study on Clinical Characteristics and Genetic Markers

**DOI:** 10.3390/genes15040473

**Published:** 2024-04-09

**Authors:** Luz María Torres-Espíndola, Juan Carlos Pérez-De Marcos, Manuel Castillejos-López, Liliana Velasco-Hidalgo, Rocío Cárdenas-Cardós, Armando De Uña-Flores, Citlaltepetl Salinas-Lara, Silvia Caballero-Salazar, Rosario Fernández-Plata, Arnoldo Aquíno-Gálvez

**Affiliations:** 1 Pharmacology Laboratory, National Institute of Pediatrics, Mexico City 04530, Mexico; pdm.jc31018@gmail.com; 2 Oncology Service, National Institute of Pediatrics, Mexico City 04530, Mexico; lilianavh@hotmail.com (L.V.-H.); oncoped_inp@hotmail.com (R.C.-C.); 3 Red MEDICI, FESI UNAM, Tlalnepantla Edo, Mexico City 54090, Mexico; 4 Hospital Epidemiology and Infectology Unit, National Institute of Respiratory Diseases Ismael Cosío Villegas, Mexico City 14080, Mexico; mcastillejos@gmail.com (M.C.-L.); rosferpla@gmail.com (R.F.-P.); 5 Radiology and Imaging Service, National Institute of Pediatrics, Mexico City 04530, Mexico; de2nails@gmail.com; 6 Department of Neuropathology, National Institute of Neurology and Neurosurgery, Manuel Velasco Suárez, Mexico City 14269, Mexico; citlalsalinas69@gmail.com; 7 Experimental Oncology Laboratory, National Institute of Pediatrics, Mexico City 04530, Mexico; caballerosalazars@gmail.com; 8 Molecular Biology Laboratory, Pulmonary Fibrosis Department, National Institute of Respiratory Diseases Ismael Cosío Villegas, Mexico City 14080, Mexico

**Keywords:** *ABC* gene, mortality, SNV, pediatric patients, central nervous system tumors

## Abstract

Multidrug resistance (MDR) commonly leads to cancer treatment failure because cancer cells often expel chemotherapeutic drugs using ATP-binding cassette (ABC) transporters, which reduce drug levels within the cells. This study investigated the clinical characteristics and single nucleotide variant (SNV) in *ABCB1*, *ABCC1*, *ABCC2*, *ABCC4*, and *ABCG2*, and their association with mortality in pediatric patients with central nervous system tumors (CNST). Using TaqMan probes, a real-time polymerase chain reaction genotyped 15 SNPs in 111 samples. Patients were followed up until death or the last follow-up day using the Cox proportional hazards model. An association was found between the rs1045642 (*ABCB1*) in the recessive model (HR = 2.433, 95% CI 1.098–5.392, *p* = 0.029), and the ICE scheme in the codominant model (HR = 9.810, 95% CI 2.74–35.06, *p* ≤ 0.001), dominant model (HR = 6.807, 95% CI 2.87–16.103, *p* ≤ 0.001), and recessive model (HR = 6.903, 95% CI 2.915–16.544, *p* = 0.038) significantly increased mortality in this cohort of patients. An association was also observed between the variant rs3114020 (*ABCG2*) and mortality in the codominant model (HR = 5.35, 95% CI 1.83–15.39, *p* = 0.002) and the dominant model (HR = 4.421, 95% CI 1.747–11.185, *p* = 0.002). A significant association between the ICE treatment schedule and increased mortality risk in the codominant model (HR = 6.351, 95% CI 1.831–22.02, *p* = 0.004, HR = 9.571, 95% CI 2.856–32.07, *p* ≤ 0.001), dominant model (HR = 6.592, 95% CI 2.669–16.280, *p* ≤ 0.001), and recessive model (HR = 5.798, 95% CI 2.411–13.940, *p* ≤ 0.001). The genetic variants rs3114020 in the *ABCG2* gene and rs1045642 in the *ABCB1* gene and the ICE chemotherapy schedule were associated with an increased mortality risk in this cohort of pediatric patients with CNST.

## 1. Introduction

Cancer is the primary cause of death among children aged 0–19 [1]. In Mexico, it is the second leading cause of death among children aged 4 to 15 [2]. Central nervous system tumors (CNST) are the second most common childhood cancer after leukemia [3]. Multidrug resistance (MDR) genes are one of the primary reasons for the failure of cancer treatment. These genes help cancer cells expel chemotherapeutic drugs from their bodies through export proteins like ATP-binding cassette (ABC) transporters. This decreases drug levels inside the cells, leading to unsuccessful treatment [4].

The subfamilies of MDR are crucial in transporting xenobiotics and anticancer drugs [5]. Among these subfamilies, ABCB1 is a part of the MDR family and is responsible for facilitating the efflux of chemotherapeutic drugs from cancer cells. On the other hand, ABCC1 and ABCG2 are responsible for transporting xenobiotics, particularly anticancer drugs. Moreover, ABCG2 can influence stem cell biology and regulate the excretion of different drugs at the blood–brain barrier (BBB) [6]; it is widely acknowledged that an individual’s genetic makeup plays a critical role in the variability of their therapeutic response, which also hinders therapeutic molecules from penetrating the brain parenchyma. SNV can affect gene expression and function, leading to differences in metabolism and drug availability among individuals with normal and cancerous cells [7,8,9,10]. The scientific literature on prognostic markers of survival and mortality in central nervous system tumors and heritable variability in *ABC* genes is limited. However, the available data suggest that *ABCG2* is significantly associated with an increased risk of progression in primary central nervous system lymphoma [11]. Studies have shown that expression levels of rs1045642 in the *ABCB1* gene are linked to prognosis, survival, and relapse in AML patients [12]. In patients with diffuse large B-cell lymphoma, high expression is associated with shorter overall and failure-free survival [13].

The ABC-type transporters play a vital role in developing chemotherapy-resistant phenotypes in malignancies. Genotyping the polymorphic sites affecting their expression and function may help predict patients’ prognoses. This study aims to determine the association between SNV in the *ABCB1*, *ABCC1*, *ABCC2*, *ABCC2*, *ABCC4*, and *ABCG2* genes and mortality rates in pediatric patients with CNST.

## 2. Methods

### 2.1. Study Subjects

The study involved 110 pediatric patients who met the following criteria: being aged between one month and 17 years, male and female, and treated for central nervous system tumors in the oncology service at the National Institute of Pediatrics in Mexico City between October 2018 and March 2020. The child patients included in this study were born in Mexico. They were considered to belong to the Mexican mestizo race because their parents and grandparents did not speak indigenous languages. These patients underwent primary chemotherapy per the treatment protocol recommended by the Mexican guidelines of the Children’s Oncology [14]. Blood samples were collected 12 h after surgery, chemotherapy, or radiotherapy. Patients were excluded due to failure to complete pharmacological treatment, poor DNA quality that failed real-time PCR amplification, and withdrawal of consent.

**Figure 1:**
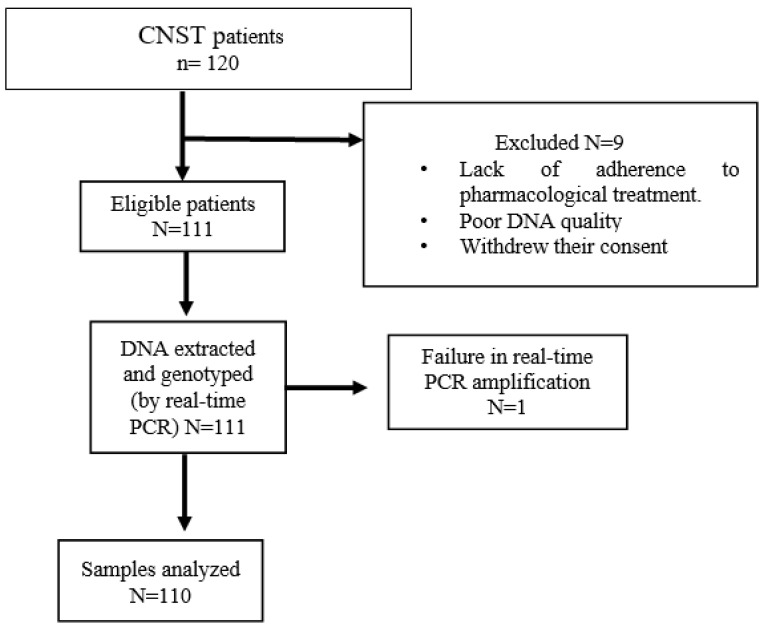
Patient enrolment flow chart.

All patients included in this study were part of a study approved by institutional committees with registration number 061/2018. The flow chart of study participants is shown in Figure 1.

Ten patients were excluded due to poor adherence to pharmacological treatment, poor DNA quality, withdrawal of consent, and failure in real-time PCR amplification.

### 2.2. Data Collection

Demographic data such as sex and age, information about tumor type, stage, and grade, type of ICE treatment vs. other non-standard treatments (ONST), and status at the end of follow-up (alive or deceased) were taken from the electronic medical record (Medsys).

### 2.3. Follow-Up (Follow-Up in Months since Initial Hospital Visit or Diagnosis)

Tumor cases were reported and tracked in a database designed for this study at the National Institute of Pediatrics in Mexico. Participants were followed from diagnosis until the end of their follow-up period, which was recorded in months.

The diagnosis of central nervous system tumors was confirmed by pathology, imaging studies, and clinical interpretation by an oncologist of electronic health records. It was coded according to the 10th International Classification of Diseases (ICD-10) Revision.

### 2.4. Selection of SNP

Candidate SNPs were selected based on frequency, response prediction, toxicity, and survival data from the MEDLINE database. The SNVs were selected based on their minor allele frequency (≥5%) and predicted functionality using SNPinfo (http://snpinfo.niehs.nih.gov/, access on 18 September 2018).

A total of fifteen probes were analyzed, including *ABCB1/MDR1* (rs1045642, rs2032582, rs1128503, rs6949448), *ABCC1/MRP1* (rs12921623, rs12921748, rs35605, rs2230671), *ABCC2/MRP2* (rs2756109, rs3740066), *ABCC4/MRP4* (rs1059751, rs4148551, rs3742106), and *ABCG2* (rs3114020, rs2231142) Table 1.

### 2.5. Genotyping

Genomic DNA was extracted from peripheral blood leukocytes using the QIAmp DNA Blood Mini kit from Qiagen, Hilden, Germany. The concentration and purity of DNA were measured using a BioTek Epoch Microplate Spectrophotometer (Agilent Technologies, Inc, Santa Clara, CA, USA), Agilent Technologies, Inc. Santa Clara, United States. Gen5 software 2.04 version, with absorbance at 260/280 nm. Integrity was evaluated via 1% agarose gel electrophoresis.

Genotyping was performed via allelic discrimination with TaqMan probes. For every SNP, three controls were used: positive (with SNP), negative (without SNP), and no template control. To prepare the reaction mixture, we combined 40 ng of gDNA, 10 pmol of each primer, 2 pmol of each probe, and 5 µL of 2X dilution master mix (provided by Applied Biosystems) in a 10 µL final volume. The thermocycling process involved 40 cycles: 30 s at 95 °C and 60 s at 60 °C. We used an Applied Biosystems Step-one instrument to read the PCR plates. To perform genotype discrimination, we used version 2.2 of the SDS software (provided by Applied Biosystems).

### 2.6. Data Analysis

The chi-square test was used to compare qualitative data, expressed as *n* (%). For quantitative data, the median and interquartile range (IQR) (Q_25_–Q_75_) were provided for both groups and compared using the Mann–Whitney U test. Patients were classified into two groups based on their status at the end of the follow-up period: survivors and deceased. Three genetic inheritance models were created—codominant (heterozygous vs. homozygous normal)/(homozygous mutated vs. homozygous normal), dominant (homozygous mutated + heterozygous vs. homozygous normal), and recessive (homozygous mutated vs. heterozygous + homozygous normal)—to evaluate the association between SNPs in *ABC* genes and survival. The Cox proportional hazards model was employed for univariate and multivariate survival analyses (adjusted model). The hazard ratio (HR) and the 95% confidence interval (95% CI) were calculated. The statistical package used for all analyses was SPSS 21.0 (Statistical Package for Social Sciences, SPSS Inc., Chicago, IL, USA). All *p*-values were bilateral, and a *p*-value < 0.05 was considered statistically significant.

## 3. Results

### 3.1. Clinical and Demographic Characteristics of the Study Population

This study analyzed 120 patients with CNST, and genotyping was done for 111 samples. The median age of the patients was 12 years (Q_25_ 6–Q_75_ 15), with the majority being males (N = 61, 55%) and females comprising N = 50, 45%. This group’s most common type of tumor was medulloblastoma (N = 31, 27.93%), followed by astrocytoma (N = 27, 24.32%). Out of the total, N = 71 (64%) had high-grade tumors, N = 75 (67.6%) were alive, and N = 36 (32.4%) were dead; the comorbidities present at diagnosis were N = 8 (7.2%), and those absent were N = 103 (92.8%) (Table 2). Headache (N = 78, 70.3%), vomiting (N = 66, 68.5%), and gait disturbance (N = 66, 59.5%) were the main symptoms in patients with tumors of the central nervous system. In addition, some patients had nausea, ataxia, decreased muscle strength, visual disturbance, weight loss, and irritability, among others Table 3.

### 3.2. Gene Frequencies in the Study Population

Table 4 shows the allele and genotype frequencies of *ABCB1*, *ABCC1*, *ABCC2*, *ABCC4*, and *ABCG2* genes in the 110 analyzed DNA samples. One patient’s DNA sample was not amplified.

### 3.3. Univariate Analysis of Allelic Variants in the ABCB1 and ABCG2 Genes Associated with Mortality

Univariate analysis for the rs1045642 variant in the *ABCB1* gene showed no association with mortality in any of the three models. For the codominant CC vs. TT model, an HR = 1.092 ((95% CI 0.472–2.526), *p* = 0.838) was obtained, and for CC vs. CT, an HR = 1.175 ((95% CI 0.486–2.842), *p* = 0.720); for the dominant model TT + CT vs. CC, an HR = 0.967 ((95% CI 0.434–2.156), *p* = 0.9350 was obtained, and for the recessive model TC + CC vs. TT, an HR = 0.754 ((96% CI 0.366–1.555), *p* = 0.445) was obtained.

For the rs3114020 variant in the *ABCG2* gene, univariate analysis showed a significant association with mortality in the three models. For the codominant CC vs. TT model, an HR of 2.9 ((95% CI = 1.094–8.071), *p* = 0.046) was obtained, and for CC vs. CT, an HR of 3.361 ((95% CI = 1.274–8.862), *p* = 0.014); for the case of the dominant model, we compared patients with the TT+TC vs. CC genotype, obtaining an HR of 3.287 ((95% CI 1.336–8.091), *p* = 0.010), and finally, for the recessive model, TC + CC vs TT, we obtained an HR of 1.16 ((95% CI 0.554–2.450), *p* = 0.010).

### 3.4. Multivariate Analysis to Estimate the Contributions of Clinical and Genetic Factors to Mortality

A multivariate analysis was performed to estimate the contribution of genetic polymorphisms adjusted by sex, age, ICE scheme, and radiotherapy to mortality. Please refer to Table 5 for results. An association was found between the SNV rs1045642 (*ABCB1*) in the recessive model (TT vs. CT + CC) with HR = 2.433 (95% CI 1.098–5.392; *p* = 0.029) and the ICE scheme in the codominant model (CC vs. TT), with an HR = 9.810 (95% CI 2.74–35.06; *p* ≤ 0.001); in the dominant model (TT + TC vs. CC), with an HR = 6.807 (95% CI 2.87–16.103; *p* ≤ 0.001); and in the recessive model (TT vs. TC + CC), with an HR = 6.903 (95% CI 2.915–16.544; *p* = 0.038), which significantly increased mortality in this cohort of patients.

An association was also observed between the variant rs3114020 (*ABCG2*) and mortality in two models: the codominant model (CC vs. TC genotype), with an HR = 5.35 (95% CI 1.83–15.39, *p* = 0.002), and the dominant model (TT + CT vs. CC genotype), with an HR = 4.421 (95% CI 1.747–11.185, *p* = 0.002).

The analysis showed a significant association between the ICE treatment schedule and increased mortality risk in all three inheritance models. The codominant model (CC vs. TT) had an HR = 6.351 (95% CI 1.831–22.02); *p* = 0.004); the CC vs. TC had an HR = 9.571 (95% CI 2.856–32.07; *p* ≤ 0.001); the dominant model (TT + TC vs. CC) had an HR = 6.592 (95% CI 2.669–16.280; *p* ≤ 0.001), and recessive model (TT vs. TC + CC) had an HR = 5.798 (95% CI 2.411–13.940; *p* ≤ 0.001).

No significant association was found between mortality and the other allelic variants in the *ABC* genes.

## 4. Discussion

In the present study, we genotyped 15 SNPs in the *ABCB1*, *ABCC1*, *ABCC2*, *ABCC4*, and *ABCG2* genes in patients with CNST. The results show that SNV rs3114020-T of the *ABCG2* gene and rs1045642-T of the *ABCB1* gene were associated with mortality.

In our study, the SNV rs1045642-T was significantly associated with mortality. According to a study conducted by Orlandi A. et al. (2018), patients with epilepsy who did not respond to drugs had a higher incidence of the TT genotype, supporting the hypothesis that the impact of *ABCB1* polymorphisms on the efficacy of antiepileptic drugs is complex and variable among different ethnic groups [15]. These results are consistent with those obtained in this work, since an association was observed between the rs1045642 and mortality.

This variant has been linked to the altered activity of the *ABCB1* multidrug resistance gene [16], pharmacoresistance in temporal lobe epilepsy [17], the risk for chronic myeloid leukemia, the atorvastatin treatment response, steroid-resistant nephrotic syndrome, prognosis in esophageal squamous cell carcinoma patients treated with a taxane [18], and clinical predictors of ondansetron failure in a diverse pediatric oncology population [19].

Olarte I. et al. (2021) reported that, in adult patients with acute myeloblastic leukemia (AML), the TT genotype rs1045642 in *ABCB1* was associated with shorter survival than the CT and CC genotypes (OR: 2.7; 95% CI: 1.28–5.81, *p* = 0.001) [20], similar to what we observed in this study.

Xiaohui S et al. studied patients with osteosarcoma. They reported that patients with the TT genotype had a higher risk of osteosarcoma death than those with the wild-type genotype (HR: 2.58 95% CI 1.03–7.28, *p* = 0.04) [21]. On the other hand, Drain S et al., in 2009, observed a shorter survival in homozygous patients with alleles (TT) with myeloma (*p* = 2 × 10^−2^) [22]. However, Zmorzynski S. et al. (2021) found that T alleles (CT and TT genotypes) in patients with multiple myeloma (MM) may be associated with a lower risk of death in patients with MM [23].

It is important to note that a high expression of *ABCB1* in tumor cells is associated with poor prognosis and the development of multidrug resistance in a way that makes them attractive prognostic markers with high clinical impact in different types of cancer in adults [24,25,26,27].

Another essential transporter is *ABCG2*, which functions as an efflux pump for drugs. Its activity is associated with the reduced efficacy of anticancer drugs in several types of cancer and, consequently, multidrug resistance; it exports drugs to the capillary lumen, preventing them from crossing the blood-brain barrier [28,29]. Our study observed an SNV rs3114020-T in the ABCG2 gene associated with mortality. *ABCG2* allelic variants are associated with different protein activities, drug sensitivities depending on the cell type and the progression and prognosis of lung cancer, leukemia, and lymphoma [30], and important genetic factors for developing gout [31].

Sun J. et al. (2017) reported for the first time that genotypes of the *ABCG2* variant rs3114020 T allele were associated with a significantly increased risk of death from non-small cell lung cancer (additive model: HR = 1.25, 95% CI 1.10–1.42, *p* ≤ 0.001) [32]. These results are consistent with the findings in our study of central nervous system tumors. More information is needed regarding this allelic variant and its clinical impact worldwide. The rs3114020 variant is known to be located in intron 1 of the *ABCG2* gene, and may affect gene expression by altering *ABCG2* transcription factor-binding sites according to SNPinfo Web http://snpinfo.niehs.nih.gov/, access on 18 September 2018 [33]. Allelic variants in noncoding regions are now known to affect the altered expression of *ABCG2* mRNA [34,35].

Strong evidence links high transporter expression with cancer patient survival and mortality. High ABC expression is associated with primary central nervous system lymphoma [11], acute myeloblastic leukemia [12], diffuse large B-cell lymphoma [13], ovarian cancer [36], osteosarcomas [37], cervical cancer [38], neuroblastoma [39] and childhood sarcoma [40].

The SNV found in this resistance gene may hinder drug efflux, leading to poor tumor response and increased mortality rates. However, it is important to note that further research is required to understand the mechanisms and extent of their contribution to mortality resulting from antineoplastic drug resistance.

In this regard, The International Transporter Consortium [41] has recognized the importance of transporters in drug therapy because of two crucial aspects. The first is that increased transporter expression and activity limit the intracellular accumulation of cytotoxic agents, thus playing an essential role in MDR to chemotherapy. The second aspect is that they show broad substrate specificity; ABCG2 transports many other drugs commonly prescribed in chemotherapy.

One limitation of our study is the absence of data on MDR expression levels and pharmacological responses in patients with solid tumors. Another limitation is that we could not stratify by tumor type or histological grade due to the limited number of tumors.

The ICE chemotherapy regimen was designed as a dose-intensive cytoreductive and stem cell mobilization regimen to treat several types of solid tumors, including high-grade nervous system tumors [42]. At the National Institute of Pediatrics, the ICE scheme treats central nervous system tumors according to the Mexican Children’s Oncology Group guidelines [14]. A recent study by Mahdy A. et al. (2023) focused on pediatric patients with classical Hodgkin’s lymphoma treated with ICE, where toxicity was assessed at the end of chemotherapy cycles, and they showed an excellent response to treatment but high hematological toxicity [43]. A study conducted by Torres LM et al. in 2020 on patients with different types of solid tumors revealed that, during the 40-month follow-up, patients who received chemotherapy that included IFA (ifosfamide), such as ICE and the mixed scheme, had a lower survival rate compared to those who did not receive IFA treatment; the *p*-value was found to be <0.001 [44]. However, in these patients, it was not possible to assess toxicity, but the high mortality may likely be due to this. Another possible reason for the increase in mortality could be the brand of antineoplastic drugs, as Mexican companies manufacture the drugs used in Mexico. However, this point has not yet been explored.

## 5. Conclusions

This study demonstrated that the SNV rs3114020 in the *ABCG2* gene, rs1045642 in the *ABCB1* gene, and the ICE chemotherapy schedule were associated with an increased mortality risk in this cohort of pediatric patients with CNTS, suggesting the importance of these variants as predictive biomarkers of mortality in CNST, as well as being related to the personalization of treatment and the minimization of toxicity to antineoplastic drugs.

## Figures and Tables

**Table 1:** Information about the selected SNPs.

Gen	SNP	Location SNP	Protein	Protein Level
*ABCB1/MDR1*	rs1045642	3435C > T, Exon26	Ile1145Ile	Reduced
	rs2032582	2677C > A, Exon 21	Ala893Ser	Reduced
	rs1128503	1236C > T, Exon 12	Gly412Gly	Reduced
	rs6949448	A > G, intron	-	altered mRNA
*ABCC1/MRP1*	rs12921623	5540C > G, Intron	-	altered mRNA
	rs12921748	5522G > A, Intron	-	altered mRNA
	rs35605	c.1684T > C, Exon14	Leu562Leu	Reduced
	rs2230671	4002G > A, Exon 28	Ser1334Ser	Reduced
*ABCC2/MRP2*	rs3740066	3972C > T, Exon 28	Ile1324Ile	Reduced
	rs2756109	G1658T, Intron7	-	altered mRNA
*ABCC4/MRP4*	rs1059751	4976T > C, 3′ URT	-	Reduced
	rs4148551	311A > G, 3′ URT	-	Reduced
	rs3742106	38T > G, 3′ URT	-	Reduced
*ABCG2*	rs3114020	−15622C > T, Promotor	-	Reduced
	rs2231142	*C421A*	Gln141Lys	Reduced

**Table 2:** General characteristics of the study population.

Characteristic	N (%)
Sex	
Male	62 (55.9)
Female	49 (44.1)
Age (years)	
Median	12
Tumor type	
Medulloblastoma	31 (27.9)
Astrocytoma	27 (24.3)
Ependymoma	20 (18.0)
Germinoma	8 (7.2)
Glioma	6 (5.4)
Glioblastoma	5 (4.5)
Neuroectodermic tumor	4 (3.6)
Pineoblastoma	2 (1.8)
Neuroglial	2 (1.8)
Rhabdomyosarcoma	2 (1.8)
Teratoma	1 (1.7)
Atypical rhabdoid teratoid	1 (1.7)
Plexus carcinoma	1 (1.7)
Astroblastoma	1 (1.7)
Grade	
High	71 (64)
Low	40 (36)
Status	
Alive	75 (67.6)
Deceased	36 (32.4)
Comorbidity	
Present	8 (7.2)
Absent	103 (92.8)

**Table 3:** The common symptoms of CNST.

Symptom	N	%
Headache	78	70.3
Threw up	76	68.5
Gait disturbance	66	59.5
Nausea	45	40.5
Ataxia	44	39.6
Decreased muscle strength	36	32.4
Visual disturbance	35	31.5
Weightless	22	19.8
Irritability	18	16.2
Behavior changes	15	13.5
Paresthesia	15	13.5
Convulsive crisis	14	12.6
Language alteration	11	9.9
Papilledema	11	9.9
Decreased sensitivity	8	7.2
Cognitive changes	8	7.2
Hemiplegia	7	6.3
Dyskinesias	6	5.4
Lymphadenopathy	4	3.6

**Table 4:** Genotype distributions and allele frequencies of *ABC* genes.

Gene	Genotypic Frequency N = (%)	Allelic Frequency
*ABCB1*		
rs1045642	N = 110	
CC	31 (30)	C = 0.52
CT	52 (50)	
TT	27(20)	T = 0.48
rs2032582	N = 110	
CC	37 (34)	C = 0.56
CA	49 (44)	
AA	24(22)	A = 0.44
rs1128503	N = 110	
CC	25 (23)	C = 0.50
CT	59 (54)	
TT	26 (23)	T = 0.50
rs6949448	N = 110	
CC	37 (34)	C = 0.57
CT	51(46)	
TT	22 (20)	T = 0.43
*ABCC1*		
rs12921623	N = 110	
GG	34 (30)	G = 0.56
GC	55 (50)	
CC	21 (20)	C = 0.44
rs12921748	N = 108	
GG	33 (30)	G = 0.56
GA	54 (50)	
AA	21(20)	A = 0.44
rs35605	N = 110	
CC	68 (62)	C = 0.78
CT	36 (33)	
TT	6 (5)	T = 0.22
*ABCC2*		
rs2756109	N = 107	
TT	24 (22)	T = 0.51
TG	62 (58)	
GG	21 (20)	G = 0.49
rs3740066	N = 110	
CC	47 (43)	C = 0.65
TC	50 (45)	
TT	13 (12)	T = 0.35
*ABCC4*	N = 110	
rs1059751		
TT	44 (40)	T = 0.67
TC	59 (54)	
CC	7 (6)	C = 0.33
rs4148551	N = 110	
TT	26 (24)	T = 0.48
CT	54 (49)	
CC	30 (27)	C = 0.52
rs3742106	N = 110	
AA	32 (29)	A = 0.52
AC	50 (45)	
CC	28 (26)	C = 0.48
*ABCG2*	N = 105	
rs3114020		
CC	43 (41)	C = 0.66
CT	54 (51)	
TT	8 (8)	T = 0.34
rs2231142	N = 105	
GG	52 (50)	G = 0.70
GT	44 (42)	
TT	9 (8)	T = 0.3
rs2230671	N = 110	
GG	49 (45)	G = 0.64
AG	43 (39)	
AA	18 (16)	A = 0.36

**Table 5:** Multivariate analysis of rs1045642 variants in *ABCB1* and rs3114020 in *ABCG2* associated with mortality risk.

Gene	Codominant				Dominant		Recessive	
*ABCB1*	HR (95% CI)	*p*-value	HR (95% CI)	*p*-value	HR (95% CI)	*p*-value	HR (95% CI)	*p* value
	CC vs. TT		CC vs. TC		TT + TC vs. CC		TT vs. TC + CC	
rs1045642	0.853 (0.331–2.203)	0.743	1.456 (0.543–3.901)	0.455	1.009 (0.441–2.310)	0.983	2.433 (1.098–5.392)	0.029 *
Sex	0.466 (0.177–1.226)	0.122	0.474 (0.192–1.171	0.106	0.591 (0.295–1.184)	0.138	0.409 (0.186–0.896)	0.026
Age	0.785 (0.674–0.913)	0.002	0.931 (0.838–1.033)	0.179	0.956 (0.891–1.026)	0.214	0.875 (0.786–0.974)	0.014
ICE scheme	9.810 (2.74–35.06)	<0.001 *	0.274 (0.110–0.682)	0.005	6.807 (2.87–16.103)	<0.001 *	6.903 (2.915–16.544)	0.038 *
Radiotherapy	0.092 (0.019–0.441)	0.003	NC		0.098 (0.026–0.367)	0.001	0.116 (0.027–0.501)	<0.001
*ABCG2*	CC vs. TT		CC vs. TC		TT + TC vs. CC		TT vs. TC + CC	
rs3114020	2.752 (0.951–7.964)	0.062	5.35 (1.83–15.39)	0.002 *	4.421 (1.747–11.185)	0.002 *	0.807 (0.365–1.785)	0.597
Sex	0.132 (0.036–0.488)	0.002	0.467 (0.178–1.223)	0.121	0.454 (0.219–0.942)	0.034	0.516 (0.243–1.094)	0.084
Age	0.835 (0.732–0.954)	0.008	0.979 (0.895–1.071)	0.645	0.952 (0.88–1.029)	0.215	0.939 (0.870–1.014)	0.110
ICE scheme	6.351 (1.831–22.02)	0.004 *	9.571 (2.856–32.07)	<0.001 *	6.592 (2.669–16.280)	<0.001 *	5.798 (2.411–13.940)	<0.001 *
Radiotherapy	0.588 (0.076–4.535)	0.610	NC		0.262 (0.048–1.419)	0.120	0.113 (0.025–0.520)	0.005

HR: hazard ratio. CI: confidence interval. ICE: ifosfamide + carboplatin + etoposide. NC: not calculable. * Statistical significance was calculated using a multivariate Cox regression.

## Data Availability

Data can be obtained from the corresponding author upon reasonable request.
